# Clinical Society

**Published:** 1899-06-10

**Authors:** 


### CLINICAL SOCIETY.

At the last meeting of the Clinical Society a very curious case was related by Mr. Betham Robinson which showed, among other things, how little one can foretell what may be found on opening an abdomen for the relief of symptoms of obstruction. A girl, aged seven years, was admitted into St. Thomas's Hospital with a history of a week's constipation, with occasional attacks of vomiting which had become constant, the vomited matter being dark brown in colour. She had a pinched appearance, but no marked collapse. There was acute pain in the belly about the umbilicus, but the abdomen moved with respiration and was not rigid. There was distension, particularly in the middle line above the umbilicus, and coils of small intestine were distinctly defined. There was comparative dulness on the left side, but there was no discharge from the rectum, and no lump could be felt in it. On opening the abdomen some fluid and lymph coagula escaped, and the small intestine was found so extremely distended that it had to be tapped. Then it was found that the colon was empty, and that to the left of the middle line, above the umbilicus, there was a mass which proved on examination to be a loop of small intestine, close to the cæcum, strangulated under a band formed by the tip of the vermiform appendix being adherent to a caseous mesenteric gland. This strangulation was the point at which the obstruction occurred. The cæcum and the whole of the ascending colon had retained their primitive peritoneal investment, so that they had been able to pass freely over to the left side. The appendix was separated and the intestines released, but the patient did not rally, and died shortly after the operation. No doubt the origin of the mischief was the caseous gland rather than the appendix. The fact that it was the appendix which had become attached was a mere accident, but this having happened a dangerous bridge was formed, under which in the end the small intestines became entangled.

